# Medical History of the West

**Published:** 1836

**Authors:** 


### II.—ORIGINAL.

### THE WESTERN JOURNAL.

*E Sylvis Nuncius.*

CINCINNATI, APRIL 1st, 1836.

### I. —Medical History of the West.

Many years since, the Editor of this Journal announced to the profession of the West, the intention of attempting to write a book on its Medical Topography, Climate and Diseases. After having formed this design, a variety of causes, successively, arose, to occasion a delay, that was as little anticipated by himself, as it could have been by those professional friends to whom he then communicated his intention. It would be useless to go into an enumeration of these sinister causes; but many of his readers will *guess*, that most of them grew out of the difficulties which, for many years, beset him as a public teacher. These difficulties he is happy to say are at length overcome, and he is now left free to prosecute an object, which seems to him of importance to the profession; but of such magnitude, as to render his success in its execution, after the *loss* of so many years, extremely doubtful. Nevertheless he is resolved to make the experiment; and, for collecting the requisite materials, intends to resort to traveling as the only mode on which reliance can be placed. By visiting the principal localities on the great platform, between the Lakes and the Gulph of Mexico, several important acquisitions can be made, either by direct personal observation, or by intercourse with gentlemen resident in different places. The former will consist chiefly of remarks on the Medical, Geology and Topography of those localities,—the latter, of accumulated information on the different climates and the various modifications of disease, which belong to particular places.

It is the intention of the EDITOR, to devote most of the two ensuing vacations of the *Medical Department* of the CINCINNATI COLLEGE, to this object; and that such of his readers, as reside on or near the route which he proposes to travel the *ensuing* summer, may prepare to give him such aid, as the importance of the object in which he is engaged may, in their opinion, call upon them to afford, he will here state its general direction, and enumerate the principal places which he expects to visit.

Leaving Cincinnati about the middle of May, he will pass through Madison, New Albany, Bloomington, and Terre Haute, in the state of Indiana; and thence by Vandalia and Lebanon, across the state of Illinois to St. Louis: Visiting some of the adjoining localities in the eastern part of Missouri, he intends to proceed to North Alabama, and ascending the Tennessee river, to visit Huntsville, Tuscumbia and Florence: Thence he will pass to Nashville, and traversing the state of Tennessee, proposes to reach Knoxville by the Fourth of July: Leaving that place for the north, he will pass from the Cumberland Gap to Maysville, in the state of Kentucky, when ascending the Ohio to Marietta, he will proceed to Athens, Zanesville, Granville, Newark, Lancaster, Columbus and Chillicothe, and other intermediate towns in Ohio, on his return to Cincinnati.

To all medical and other scientific gentlemen, residing on this extended route, and, especially to those who have been his pupils, or who are now the patrons of this Journal, the EDITOR would earnestly and respectfully address the request, that they will prepare themselves to afford him whatever aid may be in their power, in reference to the great object of his journey. All of them are capable of contributing to the proposed work, and all may be benefited by its execution. Let us show the truth of this remark, by an example. Autumnal Remittent Fever prevails from the shores of the Lakes to the Delta of the Mississippi, through many different climates, and over a still greater variety of soils and aspects. That it receives from these causes, as well as from the habits of the people, a great number of modifications, cannot be doubted; and no physician can hesitate to believe, that a knowledge of them, would be important to him in practice. This knowledge he cannot at present, acquire, for but few practitioners have published their observations, and these are scattered through different journals, many of which are not attainable by the majority of our physicians. Similar remarks might be made of various other interesting maladies; but the Editor believes, that enough has been said, to indicate the plan and object of the proposed work, and to awaken in all whom he addresses, a favorable anticipation of its value, provided it should be executed in a proper manner, and composed of such materials, as *may* be contributed by the physicians of the Valleys of the Mississippi and the Lakes. In respectfully asking them to place in his hands the results of their experience, the Editor desires to give this public pledge to his brethren, that he will regard it as a point of duty and honor, to preserve the name of every contributor in connexion with his contribution, so that each shall have justice done to him, while the reader will know on what authority the facts placed before him rest. Thus it will be a book *from* the physicians of the West, addressed *to* the physicians of the West—a work of reciprocal instruction—somewhat novel in the manner in which it is gotten up—limited to the diseases of the region of which it professes to treat—not dependent on the written archives of the profession for its facts, but copious in its acquisitions from cotemporary observers.

Of the pathological and therapeutic principles, with which the author will seek to imbue this history of the diseases of the West and South, he need only say, they will be substantially the same which he taught in Transylvania University, from 1823 to 1827, and at present teaches in the Cincinnati College. These are not the doctrines of any existing or preceding sect, but a selection from all the schools, without either an ambition or affectation of originality.

To render a work of this kind really valuable, it is important that on many subjects, the different contributors should be animated by the same spirit of inquiry, and make their observations, simultaneously, on some common plan. To bring this about, the Editor, begs leave to enumerate a few topics, of greatest interest.

1. All sketches of Medical Geology and Topography, should embrace, a notice of the nature of the rocks beneath, and of the soil that covers them ; the composition and temperature of the springs and wells ; the relative proportions of dry land, ponds, marshes and running waters ; the predominant vegetable productions ; the comparative elevations of plains and hills ; and the actual elevation of both, above the level of the Lakes or the Gulf of Mexico.

2. Observations on the different Climates, should present the temperature of the morning, at or before sun rise, and of the afternoon between two and three o'clock ; also, that of the night, in periods of summer epidemic diseases ; and at all seasons of the year, any remarkable changes from heat to cold, or *vice versa*. The states of the Barometer in the morning and afternoon, should be recorded in connexion with those of the Thermometer. The daily courses and variations of the wind, should be noticed at least twice, likewise the degree of obscuration or cloudiness of the atmosphere. The number of foggy mornings should be recorded, and the number of days of rain and snow, with general remarks on their quantity, when the observer is destitute of a rain guage. The electrical states of the atmosphere should receive especial attention; and every fact that can indicate the influence of thunder storms, and hurricanes on the health of the people, or on existing epidemics should be carefully recorded.

3. In connexion with the meteorological observations, some account should be given of the progress of vegetation; the health and diseases of plants; the appearance or absence of insects, and the diseases of domestic animals. In the same connexion might be placed, all remarks on the uncommon or prejudicial effects of different kinds of vegetable food, as indicating either a change in their qualities, or in the susceptibilities of man.

4. As diseases multiply in number, or undergo modifications of character, with the progress of society, a new country, where the population is still increasing in density, and new studies, arts, occupations, modes of living, fashions and manners, are successively introduced, presents an excellent opportunity for estimating their *modus operandi* and influence on the health of the people, and every physician should, therefore, be observant of their effects.

5. Closely related to these observations, are those on the peculiar temperaments and diseases of different classes of individuals, as the Anglo-American, the Creole, the Indian and the Negro; and all those whose occupations and pursuits are of a kind, that establish an idiosyncracy of *caste.*

6. The migratory disposition of the American people, and their distant removals into new climates and localities, furnish excellent opportunities in the West, for noting many important facts relative to acclimation, from a comparison of which, some valuable practical deductions might perhaps be made.

7. Our endemic summer and autumnal diseases, are well known to assume different characters, and to require variations of treatment in successive years. A faithful observation of these varieties, with a statement of all the physical circumstances which may be presumed to operate in their production, might at length, afford a solution of some difficult questions in their history.

8. The causes of the appearance and disappearance of epidemic diseases, and of the modifications which they undergo, would perhaps be illustrated, by simultaneous observations at different places, continued through a series of years.

9. Our endemic and epidemic diseases, although apparently originating in very different causes, are frequently observed to commingle in such manner, as, reciprocally, to modify each other, and every fact on this subject deserves to be recorded.

10. The relative prevalence of pulmonary consumption in the different latitudes and localities between the Lakes and the Gulph, should be ascertained.

11. Among the diseases of the West, dyspepsia and chronic hepatitis, hold in reference to their frequency, important places, and their causes and cure, do not seem to be well understood.

12. Neuralgias are common affections in the West; and additional inquiries into their supposed origin are necessary.

13. Ophthalmia is a western endemic, of which it would be interesting to discover the cause. Many facts seem to show that it arises from the same source as our autumnal fevers.

14. Finally, as the medical history of a country should embrace every thing that relates to the prominent and prevalent diseases of its people, a multitude of topics, in addition to the foregoing, should fix the attention of the contributors to such a work; and scarcely any fact, could be furnished, that would not find an appropriate place in the treatise.

As a considerable time will be required for the collection and arrangement, in connexion with his own, of these facts, the Editor proposes to give publicity in the Western Journal to any of them, that seem to be of immediate and special interest; and afterwards employ them as elements, in the work for which they are solicited.

To complete his researches, the Editor proposes to devote the spring and summer of 1837, to a second tour, exterior to that of the present year. It will embrace, Louisiana, Alabama,

Mississippi and Arkansas, to the south, and the basin of the Lakes to the north. To such of the readers of this Journal, as reside in the regions, then to be visited, he would at this early period, respectfully address the solicitation, that they co-operate with him in the national enterprise (if so it may be designated,) in which he is about to engage, and to the execution of which in a manner to be useful, the co-operation of his brethren of the West and South is indispensable.

